# Is Health Education at University Associated with Students' Health Literacy? Evidence from Cross-Sectional Study Applying HLS-EU-Q

**DOI:** 10.1155/2017/8516843

**Published:** 2017-10-10

**Authors:** Saulius Sukys, Vida Janina Cesnaitiene, Zbigniew Marcin Ossowsky

**Affiliations:** ^1^Department of Health, Physical and Social Education, Lithuanian Sports University, Sporto 6, LT-44221 Kaunas, Lithuania; ^2^Department of Recreation and Qualified Tourism, Gdansk University of Physical Education and Sport, K. Górskiego, 80-336 Gdańsk, Poland

## Abstract

**Background:**

Despite the large number of studies assessing health literacy, little research has been conducted with young adults. Since health literacy is related to the setting in which health information is provided, our study aim was to measure health literacy competencies in a sample of university students and to evaluate the relationships between these competencies and their university health education.

**Methods:**

A total of 912 university students (aged 18–24 years) completed the 47-item European Health Literacy Survey Questionnaire (HLS-EU-Q47).

**Results:**

Perceived difficulties with health information were related to gender, with male students reporting significantly lower health literacy scores. Studying more health education-related subjects was associated with a higher health literacy competency, due to these students' higher rates of accessing and understanding health information in the health promotion domain.

**Conclusion:**

Health literacy among young adult university students is insufficient. The subjects they study are related to their university health education; in particular, the number of health-related subjects they study is positively related to students' health promotion domain-based competencies.

## 1. Introduction

In the modern world, population welfare is a major health factor. Although health is highly dependent on an efficient healthcare system, relevant empowerment of the population to meet complex health-related demands is especially important [1]. The capacity to meet these demands is related to health literacy, which is characterized as having the motivation, knowledge, and competence to access, understand, appraise, and apply information in everyday life in order to make judgments and decisions about one's healthcare, disease prevention, and health promotion, as well as to maintain and promote quality of life throughout one's life course [2].

Over the past decade, a number of studies assessing population health literacy across different regions have been conducted [3–10]. Disparities in population-based health literacy in different countries are unsurprising, but it is worth mentioning that about 12.4% of individuals aged 15 years and older have insufficient health literacy, and almost half of the individuals surveyed (47.6%) have limited (problematic or inadequate) health literacy [5, 9]. This is important, because lower health literacy is typical among individuals who exhibit poor health, rate their health quality as low [6, 9], have worse self-management behavior [11, 12], and are less engaged in health-promoting behaviors [13].

Most studies have revealed a positive correlation between health literacy and social status [6, 9]. Yet, we do not yet have clear answers from assessing health literacy based on gender: some studies have shown that women have higher health literacy [5], while in other studies gender differences are not found [3, 7, 14]. Less ambiguous data have shown that age is negatively correlated with health literacy, with older adults having inadequate or problematic health literacy [3, 5, 7, 9, 15]. However, some studies do suggest that health literacy increases with age [8]. Although researchers often divide participants into age groups, greater attention has been given to health literacy among older adults, and the field has focused less on health literacy among young adults.

Recently, more focus has been dedicated to health literacy among children and adolescents, as was highlighted at the 3rd European Health Literacy Conference in 2015. However, like adolescence, young adulthood is often accompanied by significant growth, development, and life challenges. Having acquired adult rights and privileges, young adults often lose support from institutions and safety net programs that serve adolescents [16]. It has been observed that young adults may feel that they lack health-related information and an ability to understand and use this information [17, 18]. Yet, while access to quality healthcare services during young adulthood remains challenging [16], an ability to find, understand, and use health-related information remains important.

Despite the paucity of research on health literacy among young adults, the available evidence suggests that health literacy among those under 25 years of age is not higher than that among senior groups [5]. In addition, population studies carried out in different countries show varying health literacy results among young adults [3, 14]. Young adulthood is also particularly significant because most young people graduate from school and begin a different way of life, often including taking on studies at institutions of higher education. Most studies have revealed a positive correlation between health literacy and education [3, 4, 6, 9, 15, 19, 20], and the importance of assessing young adults is emphasized by a few studies revealing that their health literacy is inadequate [21–23]. The role of health educators, librarians, and other professionals in promoting students' skills in finding and understanding health information has been emphasized [21]. Health literacy is also related to the setting in which health information is provided [6], the education programs that teach individuals health information [24], and the academic courses students study [25], raising the question of how university health education is related to students' health literacy. We hypothesized that enrolling in health promotion-related courses of study would be associated with higher health literacy competency among university students.

There have been a number of recent discussions in the scientific literature about health literacy measurement tools [26, 27]. The European Health Literacy Survey Questionnaire (HLS-EU-Q), recently developed by the European Health Literacy Survey Consortium (HLS-EU) [1, 5], is based on an integrated health literacy model of competencies in the processes of accessing, understanding, appraising, and applying health-related information within three domains: healthcare, disease prevention, and health promotion [2]. The questionnaire approaches health literacy as a multidimensional construct and has been adapted for use in different European [1, 5, 6, 28, 29] and non-European [7, 8] countries. Although there have been attempts to develop health literacy assessment tools for young adults specifically, they remain in the pilot stage [30]. Therefore, it was reasonable for us to use the HLS-EU-Q in our research on young adults' health literacy and, in this way, contribute to the existing health literacy monitoring indicators.

This study aimed to measure health literacy among a sample of university students to answer two questions: (1) What are university students' health literacy competencies in the three health literacy domains assessed by the HLS-EU-Q? (2) To what extent does health education at university predict students' health literacy?

## 2. Materials and Methods

### 2.1. Study Population and Survey Protocol

This cross-sectional study was conducted in Lithuania during 2015 with student samples from universities in Kaunas, Klaipeda, and Vilnius. These three cities are the most highly developed centers of commerce, science, and culture in Lithuania and contain the highest concentration of universities. Participants were recruited using two-stage sampling [31]. In the first stage, universities were selected for the study. Universities had to offer a program of health and physical education courses (e.g., physiotherapy, physical education, physical activity, and lifestyle) to be included in the study. Note that students enrolled in medical education programs were excluded from the study. In study programs, we have studied 1950 students. Four universities were selected, with 1150 students studied in the chosen study programs. In the second stage, the researchers contacted all first- to fourth-year students enrolled in the target programs at the four participating universities. After receiving information about the research, 151 students refused to participate. The questionnaire was explained in detail to the 999 students who agreed to participate. Students were also informed that their responses would be anonymous and confidential. Questionnaire completion took an average of 24 minutes (range: 21–30 minutes). Due to unlikely responses (*n* = 22), an excess of missing values (*n* = 30), or missing gender or HLQ-EU-Q item responses (*n* = 35), 87 questionnaires were excluded. The final sample available for analyses was *N* = 912 university students who were aged 18–24 years (M = 21.08, SD = 1.42), of whom 63.3% were male. The gender distribution did not differ significantly across years of course enrollment (*p* = 0.06).

### 2.2. Measures

#### 2.2.1. Health Literacy

The 47-item HLS-EU-Q was developed by the HLS-EU consortium [5]. The questionnaire was translated to Lithuanian by two translation experts, after which we applied backward translation into English with translation expert assistance. The translated version was checked by two professionals in medicine and public health (a university professor and a public health practitioner). We took these experts' opinions into consideration when preparing the final Lithuanian version. A pilot study was then carried out with 200 university students [32].

The HLS-EU-Q measures health literacy across three health domains: healthcare (16 items), disease prevention (15 items), and health promotion (16 items). Within each domain, questions focus on competence in accessing (i.e., the ability to seek, find, and obtain health information), understanding (i.e., the ability to comprehend health information), appraising (i.e., the ability to interpret, filter, judge, and evaluate health information), and applying (i.e., the ability to communicate and use information to maintain and improve health) health-related information. Questions were generally phrased: “On a scale from very easy to very difficult, how easy would you say it is to: …” and response options were on a four-point Likert-type scale, where 1 means very difficult, 2 means fairly difficult, 3 means fairly easy, and 4 means very easy. We also included a fifth response option, “don't know,” which was coded as a missing value. Questionnaires with missing health literacy values (*n* = 20) were excluded from analyses, as previously mentioned.

Consistent with the original instrument, health literacy scores were standardized on a metric from 0 to 50 using the formula: (mean – 1) × (50/3) [5], where the mean is the average of all item responses for each participant. Four levels of health literacy were calculated based on this metric: <26 = “inadequate,” 26–33 = “problematic,” 34–42 = “sufficient,” and 43–50 = “excellent.” An overall health literacy score was calculated from the mean scores on all items. Index scores for all health literacy competencies across three health domains were standardized on a scale from 0 = “worst possible” to 5 = “best possible” using the following formula: (mean – 1) × (5/3).

#### 2.2.2. Relation of Course Studies to Health Education

Course enrollment was measured using the question, “Do you study courses related to health education?” Responses were categorized as 1 = “yes” or 2 = “no.” Students were also asked to report the number of health education-related course meetings they attended weekly from “none” to “more than five.” Information on gender and years of enrollment in the course of study was also collected.

### 2.3. Data Analysis

Cronbach's alphas were calculated to evaluate internal consistency, and an exploratory factor analysis and follow-up confirmatory factor analysis (CFA) were performed for each of the three health literacy domains. Items were loaded onto four factors, respectively, related to finding, understanding, judging, and applying health information competencies. For the CFA, a root mean square error of approximation (RMSEA) of 0.05 was taken to indicate good model fit and <0.08 to indicate acceptable model fit, and a comparative fit index (CFI) of >0.90 was taken to indicate good model fit and >0.95 to indicate very good model fit [33]. Pearson's correlation analyses were performed to examine the relations among the health literacy domains and among the domain-specific competencies.

Descriptive statistics (sum scores and subscores within each domain) were performed to answer the first research question. Multiple linear regression analyses were used to answer the second research question. All analyses were performed using IBM SPSS 23.0 statistics and AMOS version 23.0.

### 2.4. Ethical Considerations

Prior to commencing the study, approval was obtained from the university ethics committee. All enrolled students were informed of the study's purpose. Participants were also advised that the survey was anonymous and that they would receive no compensation. Verbal informed consent was obtained prior to participation.

## 3. Results

Separate exploratory factor analyses were conducted for the healthcare, disease prevention, and health promotion domains.

Four factors were identified for the healthcare domain: accessing (4 items), understanding (4 items), appraising (4 items), and applying (4 items) health-related information (Kaiser–Meyer–Olkin = 0.93, Bartlett's test of sphericity *χ*^2^ = 6143.94, *p* < 0.001); factor loadings ranged from 0.43 to 0.77 for accessing, 0.41 to 0.65 for understanding, 0.62 to 0.78 for appraising, and 0.56 to 0.82 for applying.

Four factors were identified for the disease prevention domain: accessing (4 items), understanding (3 items), appraising (5 items), and applying (3 items) health-related information (Kaiser–Meyer–Olkin = 0.92, Bartlett's test of sphericity *χ*^2^ = 5524.21, *p* < 0.001); factor loadings ranged from 0.43 to 0.77 for accessing, 0.52 to 0.88 for understanding, 0.41 to 0.84 for appraising, and 0.57 to 0.83 for applying.

Four factors were identified for the health promotion domain: accessing (5 items), understanding (4 items), appraising (3 items), and applying (4 items) health-related information (Kaiser–Meyer–Olkin = 0.92, Bartlett's test of sphericity *χ*^2^ = 6931.22, *p* < 0.001); factor loadings ranged from 0.51 to 0.81 for accessing, 0.44 to 0.67 for understanding, 0.44 to 0.83 for appraising, and 0.49 to 0.81 for applying.

All factor scores in the healthcare domain were significantly correlated with the total scale score (range: 0.48–0.69). All factor scores in the disease prevention domain were significantly correlated with the total score (range: 0.49–0.53). And all factor scores in the health promotion domain were significantly correlated with the total score (range: 0.41–0.68). Cronbach's alpha for the total score was 0.96. Cronbach's alphas for the three health literacy domains ranged from 0.87 to 0.90 (Table 1). Internal consistency scores for competencies ranged from 0.74 to 0.77 in the healthcare domain, 0.73 to 0.76 in the disease prevention domain, and 0.77 to 0.79 in the health promotion domain (Table 2).

CFA was conducted separately for the healthcare, disease prevention, and health promotion domains. In each CFA, we tested the validity of the four-factor structure, with competencies in accessing, understanding, appraising, and applying health-related information. The four-competency model was acceptable for the healthcare domain (RMSEA = 0.078, CFI = 0.90), the disease prevention domain (RMSEA = .080, CFI = 0.91), and the health promotion domain (RMSEA = 0.077, CFI = 0.92).

Over 70% of students of both genders indicated that they were enrolled in courses related to health education and attended an average of 2.05 (SD = 1.64, range = 0–5) healthcare-related course meetings per week. Among the whole sample, 6.9% had inadequate health literacy and 23.5% had excellent health literacy (Table 1). The highest health literacy index was in the healthcare domain, followed by the disease prevention and health promotion domains.

With regard to competencies in accessing, understanding, appraising, and applying health information, scores varied from 3 to 4 (translating to being perceived as “easy”); scores were highest for understanding health-related information (Table 2). The scores in Table 2 suggest that perceived difficulty varies among the health domains. Accessing information was perceived as the most difficult competency in the health promotion domain; applying information was perceived as more difficult in the disease prevention domain than in the healthcare domain.

We then analyzed the associations between the study variables and health literacy domains (Table 3). Gender was associated with general health literacy: females had significantly higher health literacy scores on all health domains. Whether students were enrolled in health-related courses was not significantly associated with health literacy. However, attending a higher number of health-related course meetings per week was positively associated with scores in the health promotion domain.

For health literacy competencies, multiple regression analyses showed a significant association between gender and accessing, understanding, appraising, and applying health information in the healthcare domain, as well as accessing, understanding, and appraising health information in the disease prevention domain (Table 4). Males had significantly lower scores, indicating that they experience greater difficulty in these domain-specific competencies. However, this association was less obvious in the health promotion domain, with the exception of understanding health information, on which females scored higher. There was no significant association between enrollment in health education-related courses and students' health literacy competencies, with the exception of accessing health information in the health promotion domain (Table 4). However, there was a positive association between accessing, understanding, and applying health information in the health promotion domain and attending more health-related course meetings per week.

## 4. Discussion

The study aim was to provide insight into health literacy among young adults attending university and to examine relations between literacy and the health education they are receiving at university. The ELS-EU-Q47 [1, 5] was successfully adapted for use in this study. Both a pilot study and the main project revealed that the instrument has good internal consistency (reliability) and confirmed the multidimensional construct of health literacy, which led us to analyze our participants' competencies in the different health literacy domains.

Fewer than 10% of these young adults had inadequate health literacy, and 23.5% had excellent health literacy. Previous studies in other European countries have shown that 10.3% of their participants had inadequate health literacy and 21.3% had excellent health literacy [5]. Although our study revealed a similar health literacy index distribution, it is important to consider participants' ages. General health literacy scores (mean = 35.5) are consistent across eight European countries, including across their subpopulations aged 25 years and younger (mean health literacy score = 35.10). Health literacy among the young adults in our study was most similar to that in Ireland [5]; lower than in Netherlands, Poland [5], and Albania [29]; and higher than in Germany, Austria, Bulgaria [5], and Japan [8].

Our results suggest that young adults' competencies are dissimilar across health literacy domains, consistent with other studies [5, 6]. Specifically, accessing information in the health promotion domain was perceived as more difficult than accessing information in the disease prevention and healthcare domains. However, applying information was perceived as more difficult in disease prevention than in the healthcare and health promotion domains.

In our study, females' health literacy was higher in all domains compared to males', in accordance with previous studies [5, 6], which might be explained by females' greater health knowledge [30]. However, when assessing health literacy competencies in different domains, gender differences are less clear. Females showed greater competence in the domains of healthcare and disease prevention, but not in applying health-related information. This might be explained by young adults generally feeling deficient not only in health-related information, but also particularly in their ability to use that information [17, 18]. This raises questions about both the availability of health-related information and how it is perceived.

Regarding the environment in which health information is provided, our second study question was about the extent to which the courses in which students were enrolled (and specifically whether the courses were health-related) would predict their health literacy. Increased health education enrollment predicted higher competencies in accessing, understanding, and applying health information. However, there was no significant association between whether students were enrolled in health-related courses and their competencies in the health literacy domains of healthcare and disease prevention. Nor was the number of health-related course meetings they attended weekly associated with students' competencies in the aforementioned domains, although this was associated with higher competency in accessing information in the health promotion domain. These results can be explained by the specificity of health literacy domains. The healthcare domain is specifically related to abilities in accessing information on medical or clinical issues, understanding and interpreting medical information, and complying with medical advice [2]. Health knowledge may have had less of an impact on this domain because of our sample's age, as younger age is associated with higher health literacy [3, 5, 7, 9, 15, 29]. In addition, health education is related to the promotion of active recreation, such as health-enhancing physical activity. Specific attention should be paid to those enrolled in courses related to physical education, physical activity, and lifestyle, in which the role of physical activity in health promotion is emphasized. Knowledge about health-promoting physical activity may further develop students' capacity to look for, understand, and apply knowledge about personal healthcare through more rigorous activities. This would explain why an increase in students' enrollment in health education courses is associated with better competencies in the health promotion domain, which includes the ability to find out about activities that are beneficial for one's own mental well-being, find information on healthy activities such as exercise, and take part in activities that improve health, such as joining an exercise class or sports club. Interestingly, previous studies have shown that higher health literacy is related to lower rates of exercise [5] and reduced self-reported likelihood of meeting physical activity guidelines [11, 34]. On the other hand, such results are not surprising given that recreational physical activity is frequently associated with a variety of positive health indicators [35].

Information about university students' health education, and particularly their physical activities, raises questions for future research with this age group. Studying at a university has traditionally been associated with engaging in sports activities and joining university teams. Recreational activity is also often associated with various positive health indicators [35], while competitive sports may be associated with a variety of mental and physical health problems (e.g., psychological and general health) [36, 37]. Although daily physical activity has been found to be unrelated to health literacy [5, 11, 34], correlations between participation in competitive sport and health literacy have not been studied at all. Since athletes are faced with the risk of various health problems associated with psychological stress and injuries [36, 37], which may require professional medical support [36], we hypothesize that, contrary to recreational physical activity, involvement in competitive sports would be positively related to health literacy.

Our study was not without limitations. We used a self-report instrument, which does not objectively assess true health literacy skills. In addition, a cross-sectional design cannot demonstrate a causal link between university health education and health literacy. Further, we did not collect detailed health education content, which will be important in future research. Finally, we collected limited demographic and socioeconomic sample characteristics (i.e., gender, age, and years of course enrollment).

## 5. Conclusions

This study extends our understanding of young adult health literacy and shows that health literacy rates among young adults at university are insufficient. These findings suggest that health literacy competencies vary across different health domains, indicating that young adults perceive the greatest difficulty in accessing health information within the health promotion and applying health information within the disease prevention domains. In addition, there is a positive association between enrollment in health-related university courses, and especially the number of such courses, and students' health promotion competencies.

## Figures and Tables

**Table 1:** Health literacy index.

Health literacy domain	Mean of HL index (SD)	Cronbach's *α*	Levels of health literacy (%)			
			Inadequate HL	Problematic HL	Sufficient HL	Excellent HL
General HL	35.5 (7.4)	0.96	6.9	26.2	43.5	23.5
Healthcare	36.1 (8.2)	0.90	7.4	21.8	42.7	28.1
Disease prevention	35.5 (8.6)	0.87	10.0	25.8	33.5	30.7
Health promotion	35.0 (8.7)	0.89	12.2	27.4	33.4	27.0

*Note*. HL: health literacy.

**Table 2:** Descriptive statistics per health literacy competence and domain.

Competence domain	Mean (SD)	Mean per item (SD)	Skew/kurtosis	Cronbach's *α*
Accessing	39.7 (6.2)	3.4 (0.8)	−0.24/0.01	0.84
Healthcare	12.5 (2.2)	3.5 (0.9)	−0.39/0.27	0.76
Disease prevention	12.5 (2.4)	3.6 (0.9)	−0.39/0.10	0.73
Health promotion	14.7 (3.2)	3.2 (1.1)	−0.18/0.37	0.78
Understanding	36.4 (4.9)	3.8 (0.7)	−0.56/0.10	0.81
Healthcare	12.9 (2.4)	3.7 (0.9)	−0.63/0.12	0.76
Disease prevention	10.5 (1.7)	4.1 (0.9)	−0.93/0.21	0.74
Health promotion	12.8 (2.4)	3.7 (0.9)	−0.50/0.33	0.77
Appraising	37.6 (6.2)	3.6 (0.8)	−0.30/0.32	0.86
Healthcare	12.4 (2.5)	3.5 (1.1)	−0.40/0.17	0.77
Disease prevention	15.5 (3.1)	3.5 (1.1)	−0.19/0.69	0.78
Health promotion	9.6 (2.1)	3.7 (1.1)	−0.39/0.77	0.79
Applying	34.8 (5.2)	3.6 (0.8)	−0.35/0.02	0.79
Healthcare	13.2 (2.3)	3.9 (0.9)	−0.81/0.57	0.74
Disease prevention	8.9 (2.2)	3.3 (1.2)	−0.17/0.61	0.76
Health promotion	12.7 (2.7)	3.6 (1.1)	−0.58/0.35	0.78

**Table 3:** Multiple regression analysis for the influence of demographics and study relation to health education variables on health literacy.

	General health literacy		Healthcare		Disease prevention		Health promotion	
	*β*	95% CI	*β*	95% CI	*β*	95% CI	*β*	95% CI
Gender	0.12^*∗∗*^	(0.75 to 3.03)	0.15^*∗∗∗*^	(1.19 to 3.73)	0.10^*∗∗*^	(0.48 to 3.17)	0.08^*∗*^	(−0.04 to 2.72)
Study year	−0.02	(−0.58 to 0.55)	−0.01	(−0.69 to 0.57)	0.01	(−0.61 to 0.72)	−0.03	(−0.69 to 0.64)
Studied courses related to health education	0.04	(0.78 to 2.02)	0.08	(0.17 to 2.96)	0.05	(0.67 to 2.65)	0.03	(1.05 to 2.26)
Number of health education courses per week	0.06	(−0.15 to 0.67)	0.01	(−0.41 to 0.51)	0.03	(−0.37 to 0.60)	0.12^*∗∗*^	(0.14 to 1.11)
	*R* = 0.16, *R*^2^ = 0.03^*∗∗*^		*R* = 0.17, *R*^2^ = 0.04^*∗∗∗*^		*R* = 0.12, *R*^2^ = 0.02^*∗*^		*R* = 0.15, *R*^2^ = 0.03^*∗∗*^	

*Note*. Analyses are based on mean per item; statistically significant ^*∗*^*p* < 0.05, ^*∗∗*^*p* < 0.01, and ^*∗∗∗*^*p* < 0.001.

**Table 4:** Multiple regression analysis for the influence of demographics and study relation to health education variables on health literacy competences.

Healthcare	Accessing		Understanding		Appraising		Applying	
	*β*	95% CI	*β*	95% CI	*β*	95% CI	*β*	95% CI
Gender	0.14^*∗∗∗*^	(0.07 to 0.26)	0.12^*∗∗∗*^	(0.09 to 0.24)	0.08^*∗*^	(−0.01 to 0.23)	0.15^*∗∗∗*^	(0.02 to 0.27)
Study year	−0.03	(−0.06 to 0.03)	−0.01	(−0.08 to 0.06)	−0.02	(−0.10 to 0.07)	−0.03	(−0.10 to 0.05)
Studied courses related to health education	0.06	(0.04 to 0.20)	0.07	(0.03 to 0.24)	0.07	(0.04 to 0.27)	0.06	(0.06 to 0.21)
Number of health education courses per week	0.01	(−0.03 to 0.03)	0.07	(−0.02 to 0.09)	0.01	(−0.05 to 0.06)	−0.01	(−0.05 to 0.05)
	*R* = 0.17, *R*^2^ = 0.03^*∗∗*^		*R* = 0.17, *R*^2^ = 0.03^*∗∗∗*^		*R* = 0.11, *R*^2^ = 0.01^*∗*^		*R* = 0.16, *R*^2^ = 0.03^*∗∗*^	

Disease prevention	Accessing		Understanding		Appraising		Applying	
	*β*	95% CI	*β*	95% CI	*β*	95% CI	*β*	95% CI

Gender	0.09^*∗*^	(0.02 to 0.31)	0.11^*∗∗*^	(0.04 to 0.30)	0.09^*∗*^	(−0.01 to 0.23)	0.04	(0.08 to 0.27)
Study year	0.02	(−0.07 to 0.09)	−0.01	(−0.08 to 0.06)	0.03	(−0.03 to 0.10)	−0.02	(−0.12 to 0.07)
Studied courses related to health education	0.08	(0.03 to 0.37)	0.04	(0.08 to 0.24)	0.06	(0.06 to 0.25)	0.04	(0.12 to 0.34)
Number of health education courses per week	0.05	(−0.03 to 0.09)	0.03	(−0.03 to 0.07)	0.02	(−0.04 to 0.07)	−0.03	(−0.08 to 0.05)
	*R* = 0.14, *R*^2^ = 0.02^*∗∗*^		*R* = 0.12, *R*^2^ = 0.01^*∗*^		*R* = 0.11, *R*^2^ = 0.01^*∗*^		*R* = 0.07, *R*^2^ = 0.01	

Health promotion	Accessing		Understanding		Appraising		Applying	
	*β*	95% CI	*β*	95% CI	*β*	95% CI	*β*	95% CI

Gender	0.05	(0.09 to 0.25)	0.10^*∗∗*^	(0.06 to 0.34)	0.05	(0.06 to 0.28)	0.04	( 0.08 to 0.21)
Study year	−0.04	(−0.12 to 0.05)	−0.03	(−0.10 to 0.05)	0.02	(−0.07 to 0.10)	0.04	(−0.05 to 0.11)
Studied courses related to health education	0.09^*∗*^	(−0.01 to 0.32)	0.02	(0.15 to 0.23)	0.01	(0.13 to 0.25)	0.03	(0.11 to 0.28)
Number of health education courses per week	0.11^*∗∗*^	(0.01 to 0.12)	0.11^*∗∗*^	(0.01 to 0.12)	0.06	(−0.03 to 0.10)	0.17^*∗∗∗*^	(0.04 to 0.14)
	*R* = 0.13, *R*^2^ = 0.02^*∗*^		*R* = 0.16, *R*^2^ = 0.03^*∗∗∗*^		*R* = 0.17, *R*^2^ = 0.01		*R* = 0.18, *R*^2^ = 0.04^*∗∗∗*^	

*Note*. Analyses are based on mean per item; statistically significant ^*∗*^*p* < 0.05, ^*∗∗*^*p* < 0.01, and ^*∗∗∗*^*p* < 0.001.
